# Evolutionary Dynamics of Tumor-Stroma Interactions in Multiple Myeloma

**DOI:** 10.1371/journal.pone.0168856

**Published:** 2016-12-28

**Authors:** Javad Salimi Sartakhti, Mohammad Hossein Manshaei, Soroosh Bateni, Marco Archetti

**Affiliations:** 1 Department of Electrical and Computer Engineering, Isfahan University of Technology, Isfahan, Iran; 2 School of Biological Sciences, University of East Anglia, Norwich, United Kingdom; University of Sheffield, UNITED KINGDOM

## Abstract

Cancer cells and stromal cells cooperate by exchanging diffusible factors that sustain tumor growth, a form of frequency-dependent selection that can be studied in the framework of evolutionary game theory. In the case of multiple myeloma, three types of cells (malignant plasma cells, osteoblasts and osteoclasts) exchange growth factors with different effects, and tumor-stroma interactions have been analysed using a model of cooperation with pairwise interactions. Here we show that a model in which growth factors have autocrine and paracrine effects on multiple cells, a more realistic assumption for tumor-stroma interactions, leads to different results, with implications for disease progression and treatment. In particular, the model reveals that reducing the number of malignant plasma cells below a critical threshold can lead to their extinction and thus to restore a healthy balance between osteoclast and osteoblast, a result in line with current therapies against multiple myeloma.

## Introduction

The production of growth factors is one of the most important determinants of cancer development [1]. Clones producing different growth factors can sustain each other’s growth [2, 3], and non-producer cells can rely on the growth factors diffusing from neighboring cells [4]. The production of growth factors by cancer cells, therefore, is a form of cooperation that can be studied in the framework of evolutionary game theory [5, 6].

**Fig 1:**
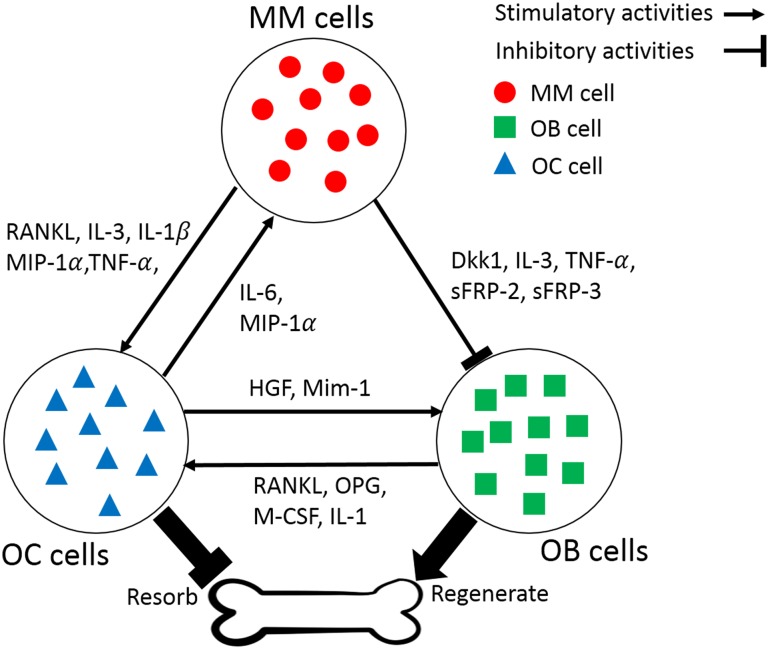
Bone remodeling in multiple myeloma. Multiple myeloma cells (MM) produce growth factors that activate osteoclasts (OC), which increase bone resorption, or that inhibit osteoblast (OB) differentiation. OC and OB secrete growth factors that affect each other and MM cells.

Cancer cells also produce diffusible factors that induce stromal cells to release other growth factors that support tumor proliferation [7]. Consider, for example, multiple myeloma, a type of cancer of plasma cells [8–10] in which different cell types—malignant plasma cells (MM) themselves, as well as osteoclasts (OC) and osteoblasts (OB)—contribute to bone resorption and bone formation by exchanging diffusible factors (Fig 1). In a healthy bone, osteoclasts demolish bone tissue and osteoblasts regenerate it, two processes that balance each other maintaining bone health. MM cells produce cytokines like interleukin-1 (IL-1), IL-3, tumor necrosis factor (TNF-α), receptor activator of nuclear factor-kB ligand (RANKL) and macrophage inflammatory protein (MIP-1α), which activate OC, and consequently increase resorption. MM cells also produce factors, such as IL-1 and Dickkopf-related protein 1 (DKK-1), which have inhibitory effects on OB differentiation. Multiple myeloma, therefore, alters the normal OB-OC equilibrium and induces, among other symptoms, bone fracture. Some factors like IL-3 have stimulatory effects on OC and inhibitory effects on OB differentiation. OC also secretes growth factors like MIP-1 and IL-6 that affect MM cell proliferation [11, 12]. In short, each cell type has stimulatory or inhibitory effects on the other types and on itself, leading to frequency-dependent selection, which can be analyzed using evolutionary game theory.

Multiple myeloma has been analyzed before in the framework of evolutionary game theory by Dingli et al. [13] using a model with pairwise interactions, that is, assuming that the effect of the growth factors produced by each cell is limited to one other companion cell. The growth factors produced by cancer and stromal cells, however, have autocrine and paracrine effects on multiple cells, therefore it would be more appropriate to model the effect of growth factors as a multiplayer game with collective interactions, rather than pairwise. While models with pairwise interactions are often used in game theory, it is known that their results cannot always be extended to games with collective interactions, and can actually lead to misleading conclusions [14].

## Model

### Fitness functions

Assume that there are *n* phenotypes in a population denoted by *P*_*1*_*…P*_*n*_. Each phenotype can produce one of *n* diffusible factors *G*_*1*_*…G*_*n*_, respectively. Each diffusible factor *j* has a different effect (*r*_i,j_) on the other phenotypes *i* (for example, *G*_*1*_ can confer a net benefit on *P*_*1*_ and *P*_*2*_, and cause a cost to *P*_*3*_). Therefore, if there are *N*_*j*_ individuals of type *P*_*j*_ among the other group members (where *N*_1_+*N*_1_+…+*N*_n_ = *N*, and *N* is the number of cells within the diffusion range of the growth factors), the payoff for strategy *P*_*j*_ is:

$$\pi_{P_{j}}(N_{1},N_{2},\ldots ,N_{n})=\frac{(N_{j}+1)c_{j}}{N}r_{j,j}+\sum_{i=1,i\neq j}^{n}\frac{N_{i}\times c_{i}}{N}r_{j,i}-c_{j}$$ (1)

Where *c*_i_ (for *i* ∈{1,…,n}) is the contribution of (that is, the cost for) *P*_i_ for growth factor *G*_i_, and *r*_i,j_ is the multiplication factor that specifies the effect of *G*_j_ on individuals belonging to type *P*_i_. For example, in a game with three phenotypes, the above equation would yield:

$$\pi_{P_{1}}\left(k,j,l\right)=\frac{\left(k+1\right)c_{1}}{N}r_{1,1}+\frac{jc_{2}}{N}r_{1,2}+\frac{lc_{3}}{N}r_{1,3}-c_{1},$$ (2)

$$\pi_{P_{2}}\left(k,j,l\right)=\frac{kc_{1}}{N}r_{2,1}+\frac{\left(j+1\right)c_{2}}{N}r_{2,2}+\frac{lc_{3}}{N}r_{2,3}-c_{2},$$ (3)

$$\pi_{P_{3}}\left(k,j,l\right)=\frac{kc_{1}}{N}r_{3,1}+\frac{jc_{2}}{N}r_{3,2}+\frac{\left(l+1\right)c_{3}}{N}r_{3,2}-c_{3},$$ (4)

Where *k*, *j*, and *l* are the numbers of individuals that invest in the growth factors *G*_1_, *G*_2_ and *G*_3_ respectively.

### Dynamics in infinite populations

In a well-mixed, infinite population we assume, as is standard, that groups are formed at random at each generation, after which fitness is calculated. Let us call *x*, *y* and *z* the frequencies of *P*_1_, *P*_2_ and *P*_3_ in the population, respectively (*x*+*y*+*z* = 1). *N* is the number of cells within the diffusion range of the growth factors, *S* is the number of individuals belonging to either type *P*_1_ or *P*_2_, and *m* is the number of individuals of type *P*_1_. Fitness is calculated by weighting the payoffs obtained in the randomly formed groups, weighted by the probability that such groups occur. The probability that a group contains *S-m-*1 individuals of type *P*_2_, *N*-*S* individuals of type *P*_3_ and *m* individuals of type *P*_1_ (in addition to the focal individual) is given by *B* (*S*-1, *N*-1, 1-*z*) × *B* (*m*, *S*-1, *x*/(1-*z*)), where:

$$B\left(x,n,p\right)=\left(\begin{array}{c} n\\ x \end{array}\right)p^{x}{\left(1-p\right)}^{n-x}$$ (5)

Therefore, the payoff for *P*_1_ is:

$$f_{1}\left(S,m\right)=\left[\frac{\left(m+1\right)c_{1}}{N}r_{1,1}+\frac{\left(S-m-1\right)c_{2}}{N}r_{1,2}+\frac{\left(N-S\right)c_{3}}{N}r_{1,3}\right]$$ (6)

and the fitness function for *P*_1_ is:

$$W_{1}=\overset{N}{\underset{S=1}{\sum }}[B\left(S-1,N-1,1-z\right)\overset{S-1}{\underset{m=0}{\sum }}B\left(m,S-1,\frac{x}{1-z}\right)\times f_{1}\left(S,m\right)]-c_{1}$$ (7)

Likewise, the fitness functions for *P*_2_ and *P*_3_ are given by:

$$W_{2}=\overset{N}{\underset{S=1}{\sum }}[B\left(S-1,N-1,1-x\right)\overset{S-1}{\underset{m=0}{\sum }}B\left(m,S-1,\frac{y}{1-x}\right)\times f_{2}\left(S,m\right)]-c_{2}$$ (8)

$$W_{3}=\overset{N}{\underset{S=1}{\sum }}[B\left(S-1,N-1,1-y\right)\overset{S-1}{\underset{m=0}{\sum }}B\left(m,S-1,\frac{z}{1-y}\right)\times f_{3}\left(S,m\right)]-c_{3}$$ (9)

where

$$f_{2}\left(S,m\right)=\left[\frac{\left(m+1\right)c_{2}}{N}r_{2,2}+\frac{\left(S-m-1\right)c_{3}}{N}r_{2,3}+\frac{\left(N-S\right)c_{1}}{N}r_{2,1}\right]$$ (10)

$$f_{3}\left(S,m\right)=\left[\frac{\left(m+1\right)c_{3}}{N}r_{3,3}+\frac{\left(S-m-1\right)c_{1}}{N}r_{3,1}+\frac{\left(N-S\right)c_{2}}{N}r_{3,2}\right]$$ (11)

The fitness functions can be reduced to:

$$W_{1}=f_{1}\left(\left(1-z\right)\left(N-1\right),x\left(N-1\right)\right)-c_{1}$$ (12)

$$W_{2}=f_{2}\left(\left(1-x\right)\left(N-1\right),y\left(N-1\right)\right)-c_{2}$$ (13)

$$W_{3}=f_{3}\left(\left(1-y\right)\left(N-1\right),z\left(N-1\right)\right)-c_{3}$$ (14)

Let us denote with *x*(*t*), *y*(*t*) and *z*(*t*) the frequencies of phenotypes *P*_1_, *P*_2_ and *P*_3_, respectively. We assume that the frequencies change according to the replicator dynamics [15]:

$$\dot{x}(t)=x(t)\left(W_{1}-\bar{W}\right),$$ (15)

$$\dot{y}(t)=y(t)\left(W_{2}-\bar{W}\right),$$ (16)

$$\dot{z}(t)=z(t)\left(W_{3}-\bar{W}\right),$$ (17)

where $\bar{W}=xW_{1}+yW_{2}+zW_{3}$. The equilibria of the system can be found by setting Equation set Eqs 15–17 equal to zero [15].

### Dynamics of diffusible factors production in multiple myeloma

We follow Dingli et al. [13] in their assumptions on the effect of each cell on another. Essentially OC and OB cells are in equilibrium in the absence of MM cells, while MM and OC cells have a stimulatory effect on each other, and MM cells inhibit OB cells and OB cells have little or no effect on MM cells [8–12, 16, 17]. Table 1 summarizes these effects, while Table 2 describes the parameters used. We can assume that *r*_*i*,*i*_ = 0 that is, each cell type has no net effect on itself. This does not mean that there are no autocrine effects; it implies, instead, that production and consumption of the growth factors are linear measures of the density of the cell types (MM cells, for instance, produce IL6 that stimulate MM cell proliferation: the more MM cells, the more IL6 is available, but also used by more MM cells). By setting *r*_*i*,*i*_ = 0 we imply that the balance changes only due to paracrine effects (in this case, the number of OC cells). *r*_MM,OB_ = 0 because *G*_OB_ (i.e., the diffusible factors produced by OB) has no effect on MM. *r*_OB,OC_ = *r*_OC,OB_ because OB and OC balance each other; for the same reasons *r*_MM,OC_ = *r*_OC,MM_.

**Table 1:** Multiplication factors for diffusible factors produced by osteoclasts (OC), osteoblasts (OB) and multiple myeloma cells (MM).

Phenotypes	Diffusible factors		
	G_OC_	G_OB_	G_MM_
OC	neutral	equilibrium	stimulation
OB	equilibrium	neutral	inhibition
MM	stimulation	neutral	neutral

**Table 2:** Multiplication factors for tumor-stroma interactions in multiple myeloma.

	Diffusible factors		
Effect on	G_OC_	G_OB_	G_MM_
OC	0	a	b
OB	a	0	-d
MM	b	0	0

Given the values in Table 2 and Eqs 12–14, the fitness of the three types of cells are:

$$W_{OC}=\;\left(bc_{3}z+ac_{2}y\right)\left(N-1\right)/N-c_{1}$$ (18)

$$W_{OB}=\;\left(ac_{1}x-dc_{3}z\right)\left(N-1\right)/N-c_{2}$$ (19)

$$W_{MM}=bc_{1}x\left(N-1\right)/N-c_{3}$$ (20)

Eqs 15–17 and 18–20 can be used to investigate the evolutionary dynamics of the three cell types over time.

## Results

Clearly, the three vertices of the simplex (*x*_OC_ = 1, *x*_OB_ = 0, *x*_MM_ = 0), (*x*_OC_ = 0, *x*_OB_ = 1, *x*_MM_ = 0) and (*x*_OC_ = 0, *x*_OB_ = 0, *x*_MM_ = 1) are fixed points of the game. Other fixed points are the three points on the edge of the simplex:

$$\{\begin{array}{c} \left(x_{OC}=\frac{ac_{2}(N-1)+(c_{2}-c_{1})N}{a(c_{1}+c_{2})(N-1)},x_{OB}=\frac{ac_{1}(N-1)+(c_{2}-c_{1})N}{a(c_{1}+c_{2})(N-1)},x_{MM}=0\right)\\ \left(x_{OC}=\frac{bc_{3}(N-1)+(c_{3}-c_{1})N}{b(c_{1}+c_{3})(N-1)},x_{OB}=0,x_{MM}=\frac{bc_{1}(N-1)+(c_{3}-c_{1})N}{b(c_{1}+c_{3})(N-1)}\right)\\ \left(x_{OC}=0,x_{OB}=\frac{dc_{3}+(c_{3}-c_{2}-c_{3}d)N}{c_{3}d(N-1)},x_{MM}=\frac{-2dc_{3}+(-c_{3}+c_{2}+2c_{3}d)N}{c_{3}d(N-1)}\right) \end{array}$$ (21)

and one in the interior of the simplex:

$$\begin{array}{l} x_{OC}=\frac{bc_{2}c_{3}N+c_{1}{}^{2}N(a-b)-c_{3}{}^{2}N(b+d)+c_{1}\{bc_{2}N+c_{3}[ab+(N-1)(b^{2}+bd)-a(b+1)N+dN]\}}{\left[a^{2}c_{1}c_{2}-abc_{1}(c_{2}+c_{3})+ac_{2}c_{3}d+bc_{1}c_{3}(b+d)](N-1\right)},\\ x_{OB}=\frac{abc_{1}c_{2}+a^{2}c_{1}c_{2}(N-1)+bc_{1}(c_{1}-c_{2})N-a[c_{1}{}^{2}+bc_{1}c_{2}+c_{2}{}^{2}-(c_{1}+c_{2})c_{3}]N}{\left[a^{2}c_{1}c_{2}-abc_{1}(c_{2}+c_{3})+ac_{2}c_{3}d+bc_{1}c_{3}(b+d)](N-1\right)},\\ x_{MM}=\frac{c_{3}[b(c_{3}-c_{2})+(c_{3}-c_{1})d]N+ac_{2}\{c_{3}[d(N-1)-N]+c_{2}N\}}{c_{3}d(N-1)} \end{array}$$ (22)

Since the fixed points and Eqs 18–20 are functions of several parameters, which makes the analysis exceedingly complex, we investigate the dynamics and the stability in three more specific scenarios. In each scenario we investigate the effect of the multiplication factors and group size on the dynamics. In what follows we show examples of the results for specific values of the parameters. Increasing the value of *a*, *b* and *d*, or reducing the value of *c*_1_, *c*_2_, *c*_3_ changes the trajectories and velocity of the dynamics but the direction and the position of the equilibria remains unaltered.

### Scenario 1

*c*_2_<*c*_1_<*c*_3_ (a common occurrence in multiple myeloma)

**Fig 2:**
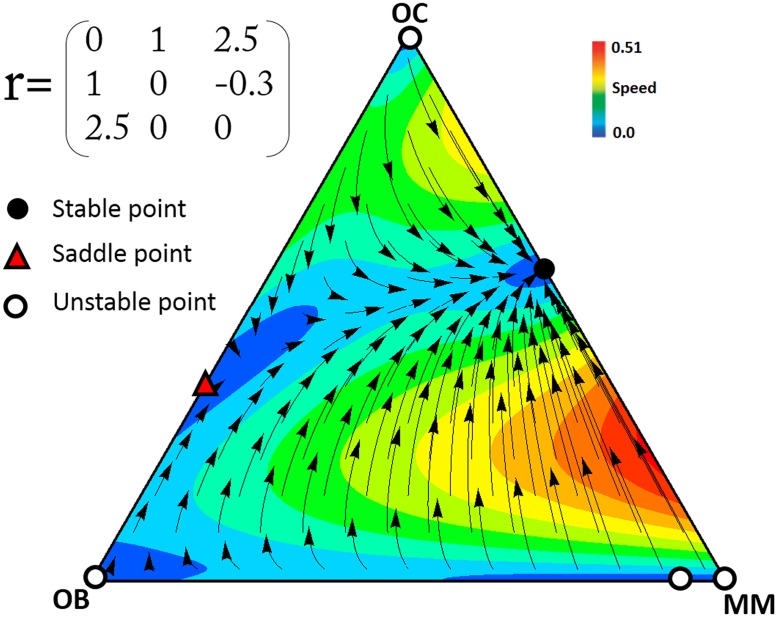
Example of the dynamics for scenario 1. In the presence of a small number of MM cells, the stable point on the OB-OC border becomes a saddle point and clonal selection leads to a stable coexistence of OC and MM cells. (*N* = 10, *c*_3_ = 1.4, *c*_2_ = 1.2, *c*_1_ = 1). The arrows show the direction of the dynamics, and the colors show its speed (the euclidean distance between the frequencies at time *t* and *t*+1).

**Fig 3:**
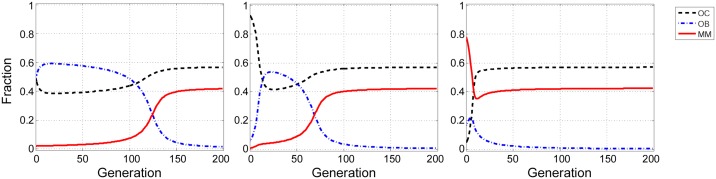
Effect of initial frequencies on the dynamics for scenario 1. Different initial frequencies of OC, OB, and MM cells do not change the final state of the population. Parameters as in Fig 2.

**Fig 4:**
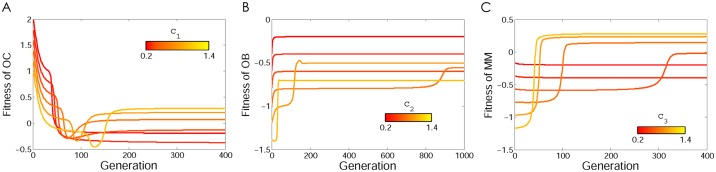
Fitness of the three cell types as a function of their contribution (cost) for scenario 1. As the value of cost increases the fitness of OC and MM increases and the fitness of OB decreases. Same parameters as Fig 2.

In multiple myeloma the contributions of the three types of cells (OC, OB, and MM) are clearly different. As mentioned above, MM cells produce growth factors and cytokines at higher levels than OB and OC cells. In the presence of MM cells, OC cells are also stimulated to produce more growth factors. Hence, it seems reasonable to study a scenario in which *c*_2_<*c*_1_<*c*_3_. Whenever the net benefit of diffusible factors that are secreted by MM cells is greater than the benefit that OC cells can obtain through the diffusible factors produced by OB, there exists a polymorphic stable point between MM and OC. Fig 2 shows an example of this scenario. While OB and OC cells are in equilibrium in the absence of MM cells, the presence of MM cells destabilizes that equilibrium and makes the population evolve to a stable mixed equilibrium of MM and OC cells, regardless of the initial frequencies (Fig 3). As the population approaches the stable point, OB cells disappear. In this process, the risk of bone fracture dramatically increases, a typical occurrence in multiple myeloma, which the model therefore explains as a perturbation of the OB-OC equilibrium by MM cells. Even a tiny fraction of MM cells in the population is able to change the dynamics and lead to an increased risk of bone fracture. Increasing the contribution (that is, the amount of diffusible factors secreted) by OC and MM cells increases their own fitness, while for OB increasing contributions decreases fitness (Fig 4).

**Fig 5:**
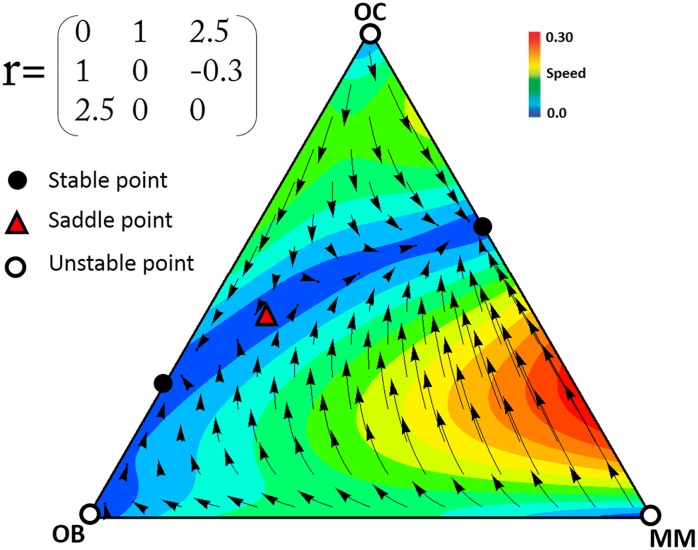
Dynamics with pairwise interactions in scenario 1. The dynamics described in Fig 2 changes when *N* = 2, resulting in a new stable point between OC and OB, and a new polymorphic saddle point, in addition to the stable point between OC and MM. The arrows show the direction of the dynamics, and the colors show its speed (the euclidean distance between the frequencies at time *t* and *t*+1).

**Fig 6:**
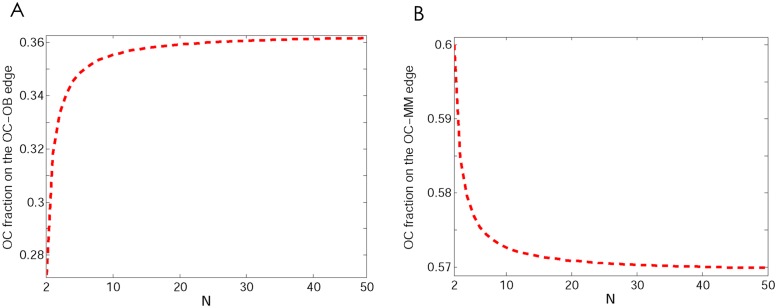
Effect of group size in scenario 1. The effect of group size *N* on the position of the fixed points on the OC-OB and OC-MM edges. Same parameters as Fig 2.

The size (*N*) of randomly composed group in our model is, essentially, the number of cells in the diffusion range of the growth factors of the focal individual. Group size has an important role in the evolutionary dynamic of the game. For example, if *N* = 2 (i.e. pairwise interaction) the dynamics described above changes and the game has two stable points on the OB-OC and OC-MM edges, and an interior saddle point (Fig 5). The presence of this interior saddle point reveals already a fundamental difference between the pairwise model [13] and our model with collective interactions (Fig 2): in our model a single MM cell is enough to lead the system from the healthy OB-OC equilibrium to the OC-MM equilibrium, whereas in the pairwise model a large number of MM cells are necessary. The effect of *N* on the position of the two fixed points of our system is described in the Fig 6.

**Fig 7:**
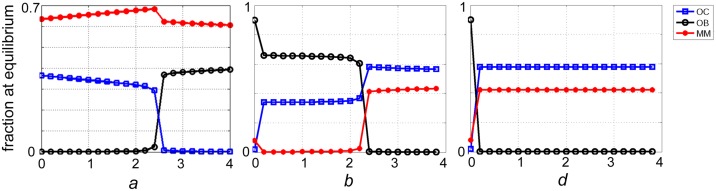
Effect of the parameters in scenario 1. The effect of *a*, *b* and *d* on the stable point in Fig 2. Changes in *a* and *b*, but not of *d*, change the stable point.

**Fig 8:**
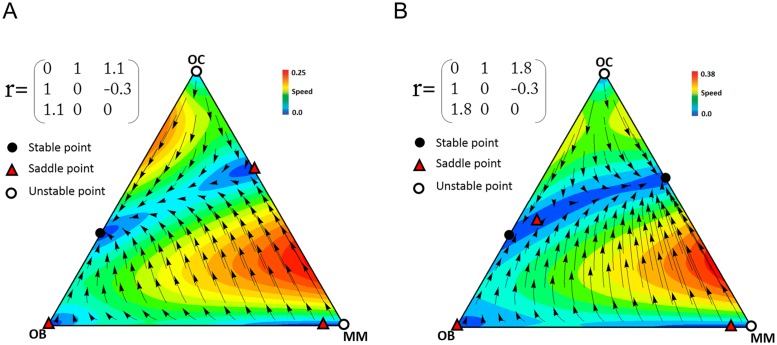
Different types of dynamics for scenario 1. (**A**) The game has one polymorphic stable point between OB and OC. In this case, clonal selection leads to the regular OC-OB balance and prevents invasion of MM cells. (**B**) The game has two polymorphic stable points. In this case, the final state of the game depends on the initial frequencies. The arrows show the direction of the dynamics, and the colors show its speed (the euclidean distance between the frequencies at time *t* and *t*+1).

In the game defined in Fig 2, when the OC and MM phenotypes coexist, their frequencies are constant with *d* (*r*_OB,MM_), and increase with *b* (where *a* has constant value 1). There may exist 3 types of stable points based on the values of *b* and *d*, a stable point between OC and MM, a stable point between OC and OB, and two stable points on the OC-MM and OC-OB (Fig 7). Fig 8 shows the two last cases. Note that MM cells (Fig 8a) are unable to modify the stable equilibrium between OB and OC cells if *b* (the effect of MM and OC on each other) is low (similar to the dynamics observed with pairwise interactions). If *b* is low enough, a further effect is that the stable mixed equilibrium of MM and OC cells turns into a saddle point.

### Scenario 2

*c*_1_ = *c*_2_ = *c*_3_ (which includes the model with pairwise interactions by Dingli et al. [13])

If the costs for OC, OB, and MM cells are equal (i.e., *c*_1_ = *c*_2_ = *c*_3_), then the following are additional fixed points:

$$\left\{ \begin{array}{l} \left(x_{OC}=\frac{1}{2},x_{OB}=\frac{1}{2},x_{MM}=0\right)\\ \left(x_{OC}=\frac{1}{2},x_{OB}=0,x_{MM}=\frac{1}{2}\right)\\ \left(x_{OC}=\frac{a^{2}-ab}{a^{2}-2ab+b^{2}+ad+bd},x_{OB}=\frac{b\left(+d-a\right)}{a^{2}-2ab+b^{2}+ad+bd},x_{MM}=\frac{ad}{a^{2}-2ab+b^{2}+ad+bd}\right) \end{array}\right.$$ (23)

In this scenario we have three type of stable points: (i) a polymorphic stable point between OC and OB, (ii) a polymorphic stable point between OC and MM and (iii) two stable points on OC-OB and OC-MM edges of the simplex.

If *a* = 1 and *N* = 2 the dynamics is equivalent to the one described by Dingli et al. [13]. *N* however is important: while *N* has no effects on the position of the fixed points, it does affect their stability (a result that is not captured by models with pairwise interactions, in which *N* = 2): if *a*<*b* and a<2*N*/(*N*-1), all eigenvalues of the Jacobean matrix are negative at (*x*_OC_ = 1/2, *x*_OB_ = 1/2, *x*_MM_ = 0), which is therefore a stable point of the system (and a healthy OC-OB balance); if *b*+*d*>*a* and *b<*2*N*/(*N*-1), all eigenvalues of the Jacobean matrix are negative at (*x*_OC_ = 1/2, *x*_OB_ = 0, *x*_MM_ = 1/2), which is therefore a stable point of the system (and an unhealthy one in which osteoblasts disappear).

**Fig 9:**
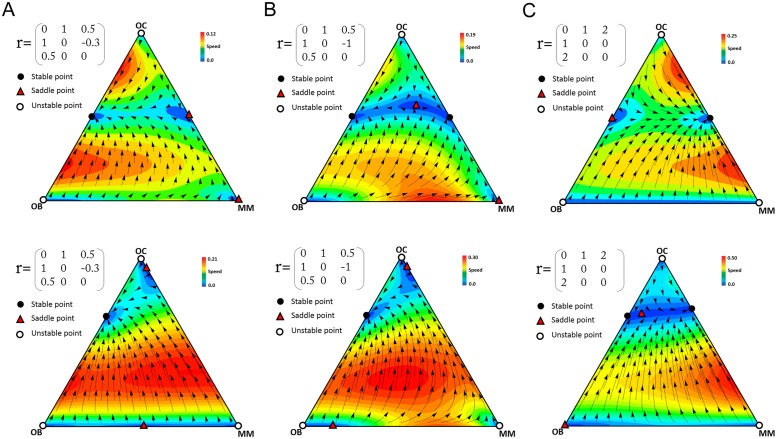
Examples of the dynamics for scenario 2. When *c*_1_ = *c*_2_ = *c*_3_ = 1, the game has (**A**) one stable point on the OC-OB edge if *a*<*b* and *a*<2*N*/(*N*-1); (**B**) two stable points on the OC-OB and OC-MM edges if *a*<*b* and *a*<2*N*/(*N*-1), *b*+*d*>*a* and *b<*2*N*/(*N*-1); (**C**) one stable point on the OC-MM edges if *b*+*d*>*a* and *b<*2*N*/(*N*-1). When the costs are not equal (bottom panels: *c*_1_ = 1, *c*_2_ = 1.2, *c*_3_ = 1.4) the dynamics and the equilibria are different. *N* = 10. The arrows show the direction of the dynamics, and the colors show its speed (the euclidean distance between the frequencies at time *t* and *t*+1).

Fig 9 shows three types of the dynamics for scenario 2. As the difference between *b* and *d* increases the stable equilibrium moves from the healthy OB-OC polymorphism to the MM-OC polymorphism typical of multiple myeloma, with a bistable system at intermediate values of the difference. Note that the cost for OC, OB, and MM changes the evolutionary dynamics at intermediate values of the b-(-d) distance (Fig 9).

### Scenario 3

*c*_3_<*c*_1_<*c*_2_ (a case with a monomorphic stable point).

**Fig 10:**
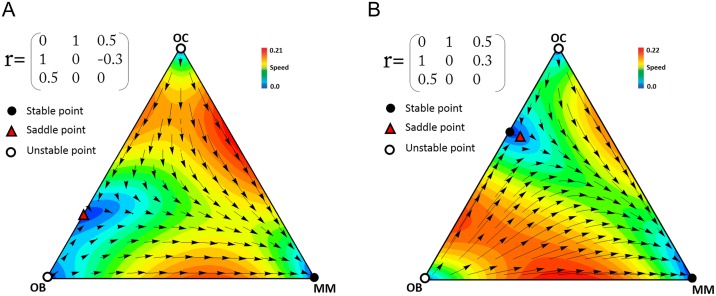
Examples of the dynamics for scenario 3. (**A**) For small group size (*N* = 10) the game has one stable point on the MM vertex. (**B**) If group size increases (*N* = 50) the game has two stable points. (*c*_1_ = 1, *c*_2_ = 1.2, *c*_3_ = 0.8). The arrows show the direction of the dynamics, and the colors show its speed (the euclidean distance between the frequencies at time *t* and *t*+1).

If *c*_3_<*c*_1_<*c*_2_, the population can have three types of stable points: a monomorphic equilibrium on the MM vertex, a polymorphic equilibrium on the OC-MM edge, and two polymorphic equilibria on the OC-MM and OC-OB edges. (The existence of the MM equilibrium can be proved analytically: if *b<*[*N*(*c*_1_*-c*_3_)]/[*c*_3_(*N-*1)] and *d*>[*N*(*c*_2_*-c*_3_)]/[*c*_3_(*N-*1)], then the MM vertex of the simplex is a stable point of the game because at (*x*_OC_ = 0, *x*_OB_ = 0, *x*_MM_ = 1) all eigenvalues of the Jacobean matrix are negative.). Fig 10 shows an example of this scenario, in which *N* affects the stable points.

### Exploiting evolutionary dynamics in response to therapy

In the three scenarios we have analysed there are polymorphic equilibria either on the OC-MM border or on the OC-OB border, or a monomorphic stable point on the MM vertex. If the only stable point of the dynamics includes at least a fraction of MM cells, clearly killing MM cells only slows down the dynamics, without leading to an effective, long-lasting treatment. However, in scenarios that include the OC-OB equilibrium, changing the parameters of the system or the fraction of cell types may affect the dynamics and change the equilibrium of the system.

**Fig 11:**
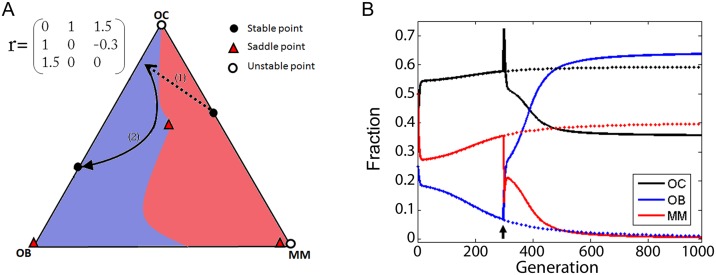
Effect of reducing the fraction of MM cells. (**A**) If a population is modified by reducing the fraction of MM cells (1, dotted arrow), it can be moved into the basin of attraction of the stable point on the OC-OB edge, and it will then evolve (2, continuous arrow) to the healthy OC-OB equilibrium. (**B**) Changes in frequencies over time corresponding to panel **A**, without therapy (dotted lines) or with therapy (continuous line) introduced at generation 300. (*c*_1_ = 1.2, *c*_2_ = 1, *c*_3_ = 1.4).

For example, consider the case in which there are two stable points, on the OC-OB and OC-MM borders (Fig 8b). It may be possible, by removing some of the MM cells, or by adding OB cells, to make the population evolve to the OC-OB mixed equilibrium, rather than to the MM-OC equilibrium (Fig 11). The same result can be achieved by changing the parameters of the game. Understanding that tumor-stroma interactions are a system with multiple equilibria, therefore, could help devise strategies to change the dynamics and make the tumor evolve to the healthy OC-OB equilibrium.

### Differences with the model of pairwise interactions

Given the linear nature of the payoffs, it is possible, in principle, to construct a game with pairwise interactions that is equivalent to our multi-player collective-interaction game, similar to what can be done for linear public goods games [18]. The payoff of the transformed pairwise game equivalent to ours is given by

$$\frac{N-1}{N}\left(\begin{array}{ccc} c_{1}r_{1,1} & c_{2}r_{1,2} & c_{3}r_{1,3}\\ c_{1}r_{2,1} & c_{2}r_{2,2} & c_{3}r_{2,3}\\ c_{1}r_{3,1} & c_{2}r_{3,2} & c_{3}r_{3,3} \end{array}\right)+\frac{1}{N}\left(\begin{array}{c} c_{1}\left(r_{1,1}-N\right)\\ c_{2}\left(r_{2,2}-N\right)\\ c_{3}\left(r_{3,3}-N\right) \end{array}\right).{\vec{1}}^{T}$$ (24)

Note that this is different from the pairwise game analyzed by Dingli et al. [13]; the core difference between their model and ours is that in our model interactions are collective (with *N*>2) rather than pairwise (*N* = 2).

**Fig 12:**
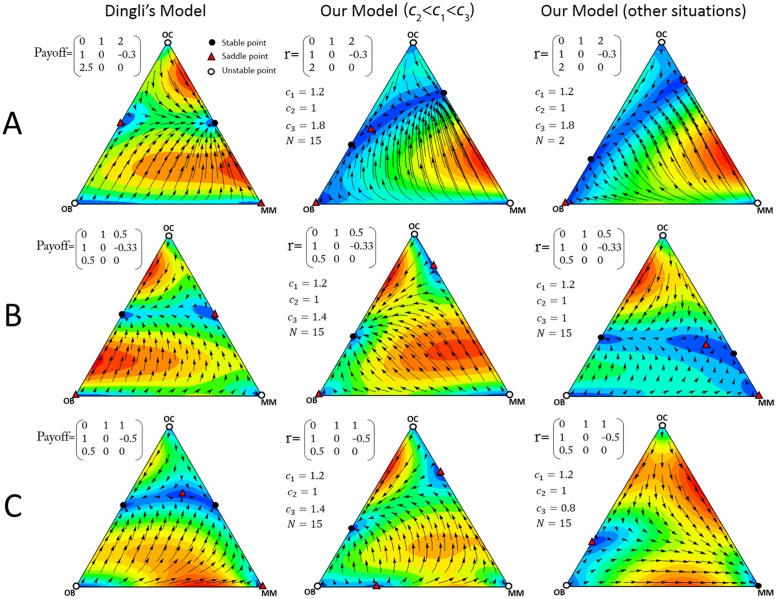
Comparison with models with pairwise interactions. A comparison of our model and the pairwise game of Dingli et al. [13] with *b*>1 (row A), *b*<1, *b+d*<1 (row B) or *b*<1, *b+d*>1 (row C). The arrows show the direction of the dynamics, and the colors show its speed (the euclidean distance between the frequencies at time *t* and *t*+1).

Dingli et al. [13], however, use the same assumptions we use about the effect of the growth factors produced by each type on each other cell type. That is, their model is equivalent to ours if we set *N* = 2, *a* = 1 and *c*_1_ = *c*_2_ = *c*_3_. These assumptions, however, seem unrealistic. In reality, not only are the costs different, but most importantly, clearly *N*>2 (that is, growth factors have a collective effect on a large number of cells, not just one cell engaged in a pairwise interaction). As we have seen, *N* has a strong effect on the stability of the fixed points. It is not surprising then, that our results (the number and nature of the equilibria) are not equivalent to the model with pairwise interactions [13] if *N*>2. Fig 12 shows some of these differences, and we discuss different cases here.

*b*>1: In this case the net benefit that OC cells obtain from MM cells is greater than what they get from OB cells. In the pairwise model of Dingli et al. [13] the system has only one stable point on the OC-MM edge and in this situation multiple myeloma eventually leads to bone fracture, due to the lack of OB cells (left panel of Fig 12a). According to our model, instead, the system can have different dynamics and stable points depending on the costs and the size of the group (that is, the diffusion range of the growth factors) (middle and right panels of Fig 12a). The differences have implications for therapy: reducing the number of MM cells in our model may help to restore a healthy OB-OC balance (see middle panel of Fig 12a), an effect that is not captured by the pairwise model of Dingli et al. [13]). In our model reducing the number of MM cells results in a recovery of the normal balance OC-OB if for any *p*_1_ and *p*_2_ (where *p*_1_*+p*_2_ = 1, *p*_1_,*p*_2_∈[0,1]):

$$4(p_{1}+p_{2}-1)\{b(c_{1}+c_{3})(N-1)p_{1}+[2c_{2}N-c_{3}d(N-1)]p_{2}\}-a(c_{1}+c_{2})(N-1)(4p_{1}p_{2}-1)>0$$ (25)

*b*<1, *b*+*d*<1: Dingli et al. [13] showed that in this case, in a pairwise model, the normal balance OC-OB is the only stable equilibrium point of the system (left panel of Fig 12b). This is the case in our model as well (middle panel of Fig 12b) if *c*_2_<*c*_1_<*c*_3_, but not if we change the values of the costs and *N* (right panel of Fig 12b). In our model, instead, an additional MM-OC stable equilibrium is possible.

*b*<1, *b*+*d*>1: In this case the pairwise model [13] has two stable points on the OC-OB and OC-MM edges (left panel of Fig 12c), while our model has different dynamics and equilibria (middle and right panels of Fig 12c). In particular, in our model the OC-MM stable equilibrium can be absent, which suggests that reducing *b*, for instance by inhibiting MIP-1*α* or IL-1*β* [19, 20] may be beneficial. This was already observed by Dingli et al. [13], and is in agreement with clinical observations [21, 22]. In our model this result is more realistic under certain parameters (middle panel of Fig 12c), as the coexistence of OB and OC can be the only stable equilibrium (whereas in the pairwise model of Dingli et al. [13], there is an additional OC-MM equilibrium, and hence the population must be pushed outside its basin of attraction); on the other hand, for different parameters (right panel of Fig 12c), a pure MM population is the only stable outcome of the dynamics.

## Discussion

As the interactions between cancer cells and stromal cells depend on the effect of diffusible factors with autocrine and paracrine effects, tumor-stroma interactions are frequency-dependent processes that can be analyzed in the framework of evolutionary game theory. Our analysis of tumor-stroma interactions in multiple myeloma departs from previous work [13] because of our assumption that cells are engaged in collective interactions rather than pairwise interactions [13]. Assuming collective interactions is more realistic, as growth factors produced by both cancer and stromal cells have autocrine and paracrine effects that are clearly not limited to just one other interacting cells; growth factors have collective effects on multiple cells, and each cell’s fitness is influenced by the production of growth factors by all the cells within the diffusion range of the factors.

As we have seen, the dynamics of tumor-stroma cooperation revealed by our model with collective interactions is more complex that observed in a model with pairwise interactions [13], and it leads to fundamental differences in the results. Like in the pairwise model analyzed by Dingli et al. [13] we observe the co-existence of two strategies when one of the three types is introduced, even at low frequency, in the population. In the presence of MM cells, the nature of the OB-OC equilibrium can change from a stable coexistence (a healthy balance of OB and OC cells) to a saddle point leading to an OC-MM equilibrium, which explains in part the pathological condition of bones observed with multiple myeloma, as the lack of OB cells, increases the risk of bone fracture [23, 24] a result also observed in the pairwise model [13].

Our results, however, show a fundamental difference. Dingli et al. [13] observe that when the net benefit that OC cells obtain from MM cells is greater than what they get from OB cells (*b*>1), which, as they point out, is generally the case [16, 25], the only stable equilibrium is the co-existence of MM and OC cells. Only if *b*<1 OB and OC cells can re-establish a stable healthy equilibrium, free from MM cells. In the pairwise model [13], in short, under realistic parameters (*b*>1) a pathological condition is inevitable when even a single MM cell is introduced in the population. In our model, instead, even with *b*>1 a reduction in the amount of MM cells can induce a change leading to a balance of OC and OB and the extinction of the MM cells (Fig 11). Additionally, the same result can be achieved, in principle, by increasing the fraction of osteoblasts.

Our model suggests, therefore, that therapies that aim at reducing the number of MM cells (for instance, protease inhibitors like Bortezomib [26–28], which is currently used in the U.S. for treating multiple myeloma) could be effective against multiple myeloma, a result that is not captured by a pairwise model [13]. In addition, it shows that extinction of malignant plasma cells could be achieved by increasing the number of osteoblasts, a cell therapy approach that might be feasible [29]. Finally, as we have seen, our model reveals that in other scenarios the dynamics of multiple myeloma can be more complex, with additional equilibria in which MM cells persist, or the collapse of MM cells and the return to a healthy balance between OB and OC cells.

Tumor-stroma interactions are, like other frequency-dependent selection processes, not intuitive, and game theory models can help understand the logic of their dynamics and, as a consequence, the feasibility of possible therapies. Our result that the malignant plasma cells may go extinct if they fall below a critical threshold required to model multiple myeloma using a model of collective interactions, which is more realistic than the assumption of pairwise interactions used previously.

Clearly one could add further realism to the model. In particular, we have assumed that the effect of the growth factors secreted by the cells is a linear function of their concentration, while the effect of growth factor is usually a nonlinear function [4, 29, 30], and it is known that non-linear benefits can lead to different dynamics. We have also assumed that cells interact at random in a well-mixed population, as if multiple myeloma was a purely liquid tumor; in reality, however, multiple myeloma could be considered in part a spatially structured population, and it is known that spatial structure can also affect the dynamics of public goods, especially with linear benefits [31]. It would be interesting to understand how nonlinearities and spatial structure in a collective action model affect the dynamics of tumor-stroma interactions.

The analysis of multiple myeloma made by Dingli et al. [13] has been pivotal in introducing game theory in the study of cooperation between tumor and the microenvironment. By extending their model to a model with collective interactions, we showed some fundamental results that a simple model with pairwise interactions could not reveal. We hope this will stimulate further analysis and further use of evolutionary game theory in the study of cancer dynamics.

## References

[1] Hanahan D. and Weinberg R. A., "The hallmarks of cancer," *cell*, vol. 100, pp. 57–70, 2000. 10647931 10.1016/s0092-8674(00)81683-9

[2] Axelrod R., Axelrod D. E., and Pienta K. J., "Evolution of cooperation among tumor cells," *Proceedings of the National Academy of Sciences*, vol. 103, pp. 13474–13479, 2006. 10.1073/pnas.0606053103 PMC1557388 16938860

[3] Cleary A. S., Leonard T. L., Gestl S. A., and Gunther E. J., "Tumour cell heterogeneity maintained by cooperating subclones in Wnt-driven mammary cancers," *Nature*, vol. 508, pp. 113–117, 2014. 24695311 10.1038/nature13187 PMC4050741

[4] Archetti M., "Heterogeneity and proliferation of invasive cancer subclones in game theory models of the Warburg effect," *Cell proliferation*, vol. 48, pp. 259–269, 2015. 25643821 10.1111/cpr.12169 PMC4964921

[5] Archetti M., "Evolutionary game theory of growth factor production: implications for tumour heterogeneity and resistance to therapies," *British journal of cancer*, vol. 109, pp. 1056–1062, 2013. 23922110 10.1038/bjc.2013.336 PMC3749558

[6] Archetti M., "Dynamics of growth factor production in monolayers of cancer cells and evolution of resistance to anticancer therapies," *Evolutionary applications*, vol. 6, pp. 1146–1159, 2013. 24478797 10.1111/eva.12092 PMC3901545

[7] Pietras K. and Östman A., "Hallmarks of cancer: interactions with the tumor stroma," *Experimental cell research*, vol. 316, pp. 1324–1331, 2010. 20211171 10.1016/j.yexcr.2010.02.045

[8] Oranger A., Carbone C., Izzo M., and Grano M., "Cellular mechanisms of multiple myeloma bone disease," *Clinical and Developmental Immunology*, vol. 2013, 2013. 10.1155/2013/289458 PMC3681224 23818912

[9] Roodman G., "Pathogenesis of myeloma bone disease," *Leukemia*, vol. 23, pp. 435–441, 2009. 19039321 10.1038/leu.2008.336

[10] Yaccoby S., "Advances in the understanding of myeloma bone disease and tumour growth," *British journal of haematology*, vol. 149, pp. 311–321, 2010. 20230410 10.1111/j.1365-2141.2010.08141.x PMC2864366

[11] Ehrlich L. A., Chung H. Y., Ghobrial I., Choi S. J., Morandi F., Colla S., et al, "IL-3 is a potential inhibitor of osteoblast differentiation in multiple myeloma," *Blood*, vol. 106, pp. 1407–1414, 2005. 15878977 10.1182/blood-2005-03-1080

[12] Lee J. W., Chung H. Y., Ehrlich L. A., Jelinek D. F., Callander N. S., Roodman G. D., et al, "IL-3 expression by myeloma cells increases both osteoclast formation and growth of myeloma cells," *Blood*, vol. 103, pp. 2308–2315, 2004. 14615378 10.1182/blood-2003-06-1992

[13] Dingli D., Chalub F., Santos F., Van Segbroeck S., and Pacheco J., "Cancer phenotype as the outcome of an evolutionary game between normal and malignant cells," *British journal of cancer*, vol. 101, pp. 1130–1136, 2009. 19724279 10.1038/sj.bjc.6605288 PMC2768082

[14] Archetti M. and Scheuring I., "Review: Game theory of public goods in one-shot social dilemmas without assortment," *Journal of Theoretical Biology*, vol. 299, pp. 9–20, 2012. 21723299 10.1016/j.jtbi.2011.06.018

[15] Hofbauer J. and Sigmund K., *Evolutionary games and population dynamics*: Cambridge University Press, 1998.

[16] Roodman G. D., "Role of the bone marrow microenvironment in multiple myeloma," *Journal of Bone and Mineral Research*, vol. 17, pp. 1921–1925, 2002. 12412796 10.1359/jbmr.2002.17.11.1921

[17] Terpos E., Sezer O., Croucher P., and Dimopoulos M.-A., "Myeloma bone disease and proteasome inhibition therapies," *Blood*, vol. 110, pp. 1098–1104, 2007. 17494860 10.1182/blood-2007-03-067710

[18] Hauert C. and Szabo G., "Prisoner's dilemma and public goods games in different geometries: compulsory versus voluntary interactions," *Complexity*, vol. 8, pp. 31–38, 2003.

[19] Dinarello C. A., "Targeting the pathogenic role of interleukin 1$\beta$ in the progression of smoldering/indolent myeloma to active disease," in *Mayo Clinic Proceedings* vol. 84, ed, 2009, pp. 105–107. 19181642 10.4065/84.2.105 PMC2664579

[20] Lust J. A., Lacy M. Q., Zeldenrust S. R., Dispenzieri A., Gertz M. A., Witzig T. E., et al, "Induction of a chronic disease state in patients with smoldering or indolent multiple myeloma by targeting interleukin 1$\beta$-induced interleukin 6 production and the myeloma proliferative component," in *Mayo Clinic Proceedings* vol. 84, ed, 2009, pp. 114–122. 19181644 10.4065/84.2.114 PMC2664581

[21] Hashimoto T., Abe M., Oshima T., Shibata H., Ozaki S., Inoue D., et al, "Ability of myeloma cells to secrete macrophage inflammatory protein (MIP)-1$\alpha$ and MIP-1$\beta$ correlates with lytic bone lesions in patients with multiple myeloma," *British journal of haematology*, vol. 125, pp. 38–41, 2004. 15015966 10.1111/j.1365-2141.2004.04864.x

[22] Terpos E., Politou M., and Rahemtulla A., "New insights into the pathophysiology and management of bone disease in multiple myeloma," *British journal of haematology*, vol. 123, pp. 758–769, 2003. 14632767 10.1046/j.1365-2141.2003.04712.x

[23] Bataille R. and Harousseau J.-L., "Multiple Myeloma," *New England Journal of Medicine*, vol. 336, pp. 1657–1664, 1997. 9171069 10.1056/NEJM199706053362307

[24] Kyle R. A. and Rajkumar S. V., "Multiple Myeloma," *New England Journal of Medicine*, vol. 351, pp. 1860–1873, 2004. 15509819 10.1056/NEJMra041875

[25] Epstein J. and Yaccoby S., "Consequences of interactions between the bone marrow stroma and myeloma," *The Hematology Journal*, vol. 4, pp. 310–314, 2003. 14502254 10.1038/sj.thj.6200313

[26] Adams J. and Kauffman M., "Development of the proteasome inhibitor Velcade^™^(Bortezomib)," *Cancer investigation*, vol. 22, pp. 304–311, 2004. 15199612 10.1081/cnv-120030218

[27] Curran M. P. and McKeage K., "Bortezomib," *Drugs*, vol. 69, pp. 859–888, 2009. 19441872 10.2165/00003495-200969070-00006

[28] Moreau P., Pylypenko H., Grosicki S., Karamanesht I., Leleu X., Grishunina M., et al, "Subcutaneous versus intravenous administration of bortezomib in patients with relapsed multiple myeloma: a randomised, phase 3, non-inferiority study," *The lancet oncology*, vol. 12, pp. 431–440, 2011. 21507715 10.1016/S1470-2045(11)70081-X

[29] Jourdan M., Mahtouk K., Veyrune J.-l., Couderc G., Fiol G., Redal N., et al, "Delineation of the roles of paracrine and autocrine interleukin-6 (IL-6) in myeloma cell lines in survival versus cell cycle. A possible model for the cooperation of myeloma cell growth factors," *European cytokine network*, vol. 16, pp. 57–64, 2005. 15809207

[30] Karey K. P. and Sirbasku D. A., "Differential responsiveness of human breast cancer cell lines MCF-7 and T47D to growth factors and 17$\beta$-estradiol," *Cancer Research*, vol. 48, pp. 4083–4092, 1988. 3289739

[31] Perc M. v., Gómez-Gardeñes J., Szolnoki A., Floría L. M., and Moreno Y., "Evolutionary dynamics of group interactions on structured populations: a review," *Journal of The Royal Society Interface*, vol. 10, p. 20120997, 2013. 10.1098/rsif.2012.0997 PMC3565747 23303223

